# Direct Molecular Action of Taurine on Hepatic Gene Expression Associated with the Amelioration of Hypercholesterolemia in Rats

**DOI:** 10.3390/antiox13080990

**Published:** 2024-08-14

**Authors:** Qi Song, Satoru Kobayashi, Yutaro Kataoka, Hiroaki Oda

**Affiliations:** Laboratory of Nutritional Biochemistry, Nagoya University, Nagoya 464-8601, Japan

**Keywords:** taurine, cholesterol, transcriptome, hepatic

## Abstract

Taurine can ameliorate hypercholesterolemia by facilitating cholesterol efflux and increasing cytochrome P450 7A1 (CYP7A1) without clear underlying molecular mechanisms. This study aims to elucidate the molecular action of taurine in diet-induced hypercholesterolemia. Male Wistar rats were fed a high cholesterol diet containing 5% taurine for 14 days. Three-dimensional primary hepatocytes from rats were exposed to 10 mM taurine for 24 h. Transcriptome analyses of both the liver and hepatocytes were performed using DNA microarray. Taurine significantly decreased serum cholesterol levels and increased hepatic CYP7A1 mRNA levels and transcription rates in rats. Taurine altered the expression of seventy-seven genes in the liver, involving lipid, drug, amino acid metabolism, and gluconeogenesis pathways. The small heterodimer partner (SHP), a transcription factor regulated by taurine, was suppressed. “Network analysis” revealed a negative correlation between the SHP and induction of CYP7A1 and cytochrome P450 8B1 (CYP8B1). However, CYP7A1 and CYP8B1 levels were not altered by taurine in 3D-primary hepatocytes. Venn diagram analyses of the transcriptomes in both hepatocytes and the liver indicated a consistent upregulation of organic anion transporting polypeptide 2 (OATP2) and betaine homocysteine methyltransferase (BHMT). Taurine ameliorated hypercholesterolemia in rats fed a high cholesterol diet by directly enhancing the hepatic expression of BHMT and OATP2, which modulated the SHP and induced CYP7A1 and CYP8B1, thereby promoting cholesterol catabolism and lowering blood cholesterol levels.

## 1. Introduction

Taurine, a non-proteinogenic β-amino sulfonic acid, is mostly found in animal-source foods (such as beef and fish) and not in terrestrial plants [1,2]. It is a non-essential nutrient that can be synthesized endogenously, mainly in the liver, from cysteine in a reaction catalyzed by cysteine dioxygenase, cysteinesulfinic acid decarboxylase, and flavin-containing monooxygenase 1 in mammals [3,4,5]. As one of the most enriched free amino acids, taurine accounts for nearly 0.01% of total human body weight. It is closely associated with the healthy lifespan of mammals because of its various cytoprotective functions, such as antioxidation, intracellular ion regulation, osmoregulation, and immunomodulation [6,7,8].

Research indicates that blood taurine concentrations decrease with cellular senescence, mitochondrial dysfunction, and inflammation during aging in mice, monkeys, and even humans [6,9]. Taurine supplementation has been shown to improve health conditions such as obesity, liver diseases, cardiovascular diseases, diabetes, and hypertension, suggesting its huge potential to counteract the aging process [6,10]. Since aging significantly affects the heart and arterial system, maintaining adequate taurine levels is also crucial for cardiovascular health [11]. Taurine alleviates the symptoms of a range of cardiovascular diseases, which remain the leading cause of death worldwide, and rapidly attenuates hypercholesterolemia among them [12,13,14]. Hypercholesterolemia is a major risk factor for cardiovascular diseases. Chen et al. demonstrated that feeding rats or mice a high cholesterol/sodium cholate diet supplemented with 1% taurine for one week resulted in a reduction in cholesterol levels by approximately 20% to 42%, with decreases in low-density lipoprotein (LDL) and very low-density lipoprotein cholesterol ranging from 26% to 66% [15]. Similarly, experimental results from our laboratory indicated the beneficial effects of taurine on exogenous hypercholesterolemia [16]. Regarding endogenous hypercholesterolemia caused by diabetes or xenobiotics, a 13–27% decrease in serum (or plasma) cholesterol was also observed with taurine [17,18]. These studies also suggested that taurine lowered cholesterol levels by promoting cholesterol efflux. The action of taurine is mainly achieved through three possible mechanisms [19]. Specifically, at the source, taurine upregulates hepatic LDL receptor binding capacity to accelerate the clearance of cholesterol from the blood [20]. At the terminal stage, taurine improves the excretion of cholesterol and bile acids from the intestine [21], whereas the most important intermediate role is the conversion of cholesterol to bile acids in the liver [16,21].

All mammalian cells produce cholesterol; however, only hepatocytes, adrenal cells, and gonadal cells can catabolize cholesterol [22]. Different from adrenal and gonadal cells that metabolize cholesterol steroid hormones, hepatocytes are able to catabolize cholesterol and facilitate its elimination from the body. Most excess cholesterol is used in conjunction with bile acid in the liver, which is critical for maintaining cholesterol homeostasis [23]. Cholesterol 7α-hydroxylase (cytochrome P450 7A1, CYP7A1), the rate-limiting enzyme in the conversion of cholesterol to bile acids, is key to the cholesterol efflux facilitated by taurine. Several studies have shown that taurine increases CYP7A1 activity and mRNA levels by approximately 2-fold in rats and mice fed a high cholesterol diet, as well as in the human liver cell line HepG2 [15,16,24,25,26]. The cholesterol-lowering effect of taurine through the enhancement of CYP7A1 was confirmed in the aforementioned studies, and the regulation of CYP7A1 has also been thoroughly investigated [27]. However, the molecular mechanism of the action of taurine on CYP7A1 has rarely been investigated. Hence, for a comprehensive investigation into the effects of taurine and the genes it regulates, the transcriptomic analysis of high cholesterol diet-fed rats and three-dimensional (3D) primary hepatocytes [28,29] was performed. Initially, we examined the role of taurine in liver gene expression and its subsequent effects on liver function within the complex in vivo environment. Subsequently, we investigated the direct impacts of taurine on the liver using in vitro primary rat hepatocyte cultures. Maintaining liver-specific phenotype and normal gene expression requires cell shape, cell–cell communication, cell–extracellular matrix contact, and cell polarity. Three-dimensional spherical hepatocytes cultured under these conditions exhibit enhanced functionality, and EHS-gel, a classic pattern widely used in our lab, preserves the differentiated state of primary hepatocytes [28,29].

Therefore, this study aims to elucidate the mechanisms of cholesterol-lowering action of taurine and its extensive physiological functions at the molecular level.

## 2. Materials and Methods

### 2.1. Animals

All animal experiments were approved by the Animal Research Committee of the Center for Animal Research and Education at Nagoya University (permission number 2008060901). All procedures were performed in strict accordance with the guidelines stipulated by the committee. The animals were housed independently at an average temperature of 23 ± 1 °C, cycled with 12 h light (08:00–20:00) and 12 h dark.

For the animal experiments, 24 five-week-old male Wistar rats (SLC, Hamamatsu, Japan) weighing approximately 60–80 g were used. After an acclimatization period of two days on a Labo MR standard solid diet (NOSAN, Kanagawa, Japan) and two days on a 20% (g/kg diet) casein powder diet, the rats were divided into groups of six rats each and fed ad libitum on the control (C group), a high cholesterol diet (H group), and their corresponding 5% taurine-containing powder diets (CT and HT groups). The compositions of the four diets are shown in Table 1. The rats were euthanized by decapitation without anesthesia after 4 h of fasting at 20:00 on day 14. Blood was immediately collected directly into test tubes, and serum was separated by centrifugation at 4500 rpm at 4 °C for 10 min after being left to clot for 15 min at room temperature. Liver and epididymal adipose tissue samples were collected, weighed, immediately frozen in liquid nitrogen, and stored at −80 °C until further analysis.

To isolate primary hepatocytes, male Wistar rats weighing approximately 180 g were obtained from SLC Japan (Hamamatsu, Japan) and fed Labo MR Standard Solid Food (NOSAN, Kanagawa, Japan).

### 2.2. Rat Primary Hepatocyte 3D Culture

Primary hepatocytes were isolated from Wistar rats using a two-step collagenase perfusion method, with quality assessed by trypan blue exclusion showing cell viability typically greater than 90% [30]. The cells were then cultured in Waymouth’s MB 752/1 medium containing penicillin (5 IU/mL) and streptomycin (5 mg/mL) following previously described procedures [29,31]. Primary hepatocytes were seeded at an initial density of 1 × 10^7^ cells/dish on Engelbreth-Holm swarm (EHS) gel, a classic 3D culture pattern that emphasizes the maintenance of hepatocyte differentiation. After 24 h, primary hepatocytes were subjected to treatment. The N group was treated with 1 × 10^−6^ M dexamethasone, whereas the NT group received 1 × 10^−6^ M dexamethasone and 10 mM taurine for 24 h.

### 2.3. Biochemical Analysis

Serum biochemical parameters (total cholesterol, triglycerides, phospholipids, and non-esterified fatty acids (NEFA)) were measured using commercial kits (Cholesterol E-test, Triglyceride E-test, Phospholipids C-test, and NEFA C-test, FUJIFILM Wako Pure Chemical Corporation, Osaka, Japan).

### 2.4. Total RNA Extraction and Quantitative Real-Time PCR Analysis (RT-qPCR)

Total RNA was extracted from the liver using the acid guanidinium thiocyanate–phenol–chloroform extraction method [32]. RNA integrity was assessed by microchip electrophoresis using MultiNA (Shimadzu, Kyoto, Japan) after DNase treatment. Next, cDNA was synthesized using ReverTra Ace^®^ qPCR RT Master Mix (TOYOBO Co., Ltd., Osaka, Japan). RT-qPCR was performed using the Power SYBR™ Green PCR Master Mix (Thermo Fisher Scientific Inc., Waltham, MA, USA) in the StepOnePlus Real-Time PCR System (Thermo Fisher Scientific). The transcription rate was determined using first intron-specific primers for premature mRNA, as previously described by Lipson and Baserga [33].

The mRNA level of apolipoprotein E (ApoE) was not affected by either the high cholesterol diet or taurine and was, therefore, used for normalization standard [16]. Primer sequences are listed in Supplementary Table S1.

### 2.5. Microarray Analysis and Data Analysis

Total RNA was extracted from the liver tissues of the H and HT groups and from the primary hepatocytes of the N and NT groups. Subsequently, the RNA was reverse-transcribed to cDNA, which was then combined and hybridized to the Agilent rat oligonucleotide microarray (Agilent Technologies, Santa Clara, CA, USA) for the H and HT groups and to the Rat Genome 230 2.0 Array (Affymetrix, Santa Clara, CA, USA) for the N and NT groups, with subsequent scanning of the microarray slides.

Data analysis of the H and HT groups was performed using GeneSpring version 7.3 (Agilent Technologies) for locally weighted scatterplot smoothing, normalization, and ratio calculations. Differences in gene expression were considered significant at *p* < 0.01. False discovery rate (FDR) adjustment was applied to control for multiple comparisons, and genes were deemed significant if the FDR was less than 0.05. For the N and NT groups, data automatization and normalization were performed using the Microarray Suite (Affymetrix), and genes showing differential expression between the NT and N groups (log2 ratio ≥ 0.5 or ≤−0.5) were screened.

Differentially expressed genes were subjected to filtered Ingenuity Pathway Analysis (IPA) with the conditions set to “Species” and “Tissues” as “Rat” and “Liver.” Subsequently, “Function” and “Pathway” analyses were conducted on the filtered differentially expressed genes using IPA version 8.5 (Ingenuity Systems, Redwood City, CA, USA), and a series of genes belonging to certain genetics networks were also identified.

The microarray data have been uploaded to DDBJ and are available under the accession numbers E-GEAD-825 and E-GEAD-826.

### 2.6. Statistical Analyses

Statistical analyses were performed using SPSS Statistics version 22.0 (IBM Corp, Armonk, NY, USA), and the values are presented as the mean ± standard error of the mean. Two-way analysis of variance (ANOVA) was employed to evaluate the main effects of a high cholesterol diet and taurine supplementation, as well as their interaction effect, across four experimental groups. Statistical significance was set at *p* < 0.05. In cases of significant interaction effects, post hoc analyses were performed using Student’s *t*-test with total residual standard error of the mean (SEM).

## 3. Results

### 3.1. Effect of Taurine on the Physiological and Biochemical Parameters in Rats Fed a High Cholesterol Diet

**Figure 1:**
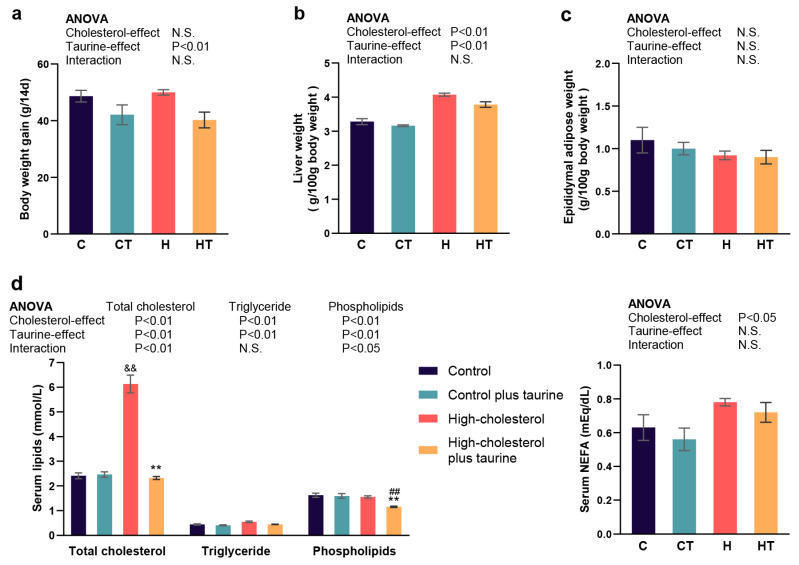
Taurine ameliorated serum cholesterol accumulation in high cholesterol diet-fed rats. (**a**) Body weight gain; (**b**,**c**) liver and epididymal adipose tissue weight; and (**d**) serum total cholesterol and triglyceride, phospholipid, and NEFA levels. C: control group, CT: control plus taurine group, H: high cholesterol group, and HT: high cholesterol plus taurine group. Values represent the mean ± standard error of the mean (SEM), n = 6 of each group. The statistical results of two-way ANOVA are indicated above each figure. *p* < 0.05 was considered significant. N.S.: not significant. When the interaction effect was significant, Student’s *t*-test was performed with residual total SEM, and significant results were labeled on the value bars in each figure. && These values differed significantly (*p* < 0.01) from the value of the control diet group. ## These values differed significantly (*p* < 0.01) from the value of the taurine diet group. ** These values differed significantly (*p* < 0.01) from the value of the high cholesterol diet group.

After two weeks of feeding, a high cholesterol diet significantly increased liver weight and serum total cholesterol, triglyceride, and NEFA levels and decreased serum phospholipid levels (Figure 1b,d). Taurine reduced body weight gain; liver weight; and serum total cholesterol, triglyceride, and phospholipid levels (Figure 1a,b,d). Furthermore, a significant interaction was observed between the high cholesterol diet and taurine on serum total cholesterol and phospholipid levels, with taurine demonstrating a more pronounced decrease in rats fed the high cholesterol diet (Figure 1d). No significant differences in adipose tissue weight were observed (Figure 1c).

### 3.2. Taurine Increased Hepatic CYP7A1 Gene Expression and Transcription Rate in Rats Fed a High Cholesterol Diet

**Figure 2:**
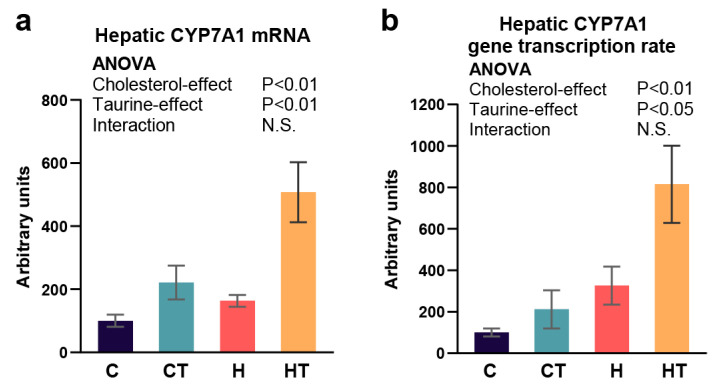
Taurine increased mRNA level and transcription of CYP7A1 gene in high cholesterol diet-fed rats. (**a**) Hepatic CYP7A1 gene expression level and (**b**) hepatic CYP7A1 gene transcription rate. C: control group, CT: control plus taurine group, H: high cholesterol group, and HT: high cholesterol plus taurine group. Values represent the mean ± SEM, n = 6 of each group. The statistical results of two-way ANOVA are indicated. *p* < 0.05 was considered significant. N.S.: not significant.

First, the previously known mechanism by which taurine improves hypercholesterolemia by increasing CYP7A1 and promoting cholesterol catabolism to bile acids was confirmed. Taurine significantly increased hepatic CYP7A1 mRNA levels and transcription rates based on the induction effect of a high cholesterol diet (Figure 2a,b). This indicates that taurine activates the gene expression of CYP7A1 at the transcriptional level.

### 3.3. “Function” and “Pathway” Analyses of Taurine Effect in Transcriptomics of Hepatic RNA in Rats Fed a High Cholesterol Diet

To investigate the effect of taurine on CYP7A1, microarray analysis was performed on liver RNA samples obtained from the high cholesterol diet and the high cholesterol diet plus taurine groups. Genes showing significant variation (*p* < 0.01) in expression due to taurine supplementation in the high cholesterol diet group were identified as differentially expressed genes, which were subsequently filtered by species “rat” and tissue “liver” using IPA filter criteria and a 1.35-fold change threshold (Supplementary Table S2). Analysis revealed that taurine supplementation increased the expression of 48 genes and decreased the expression of 29 genes (Supplementary Table S2).

“Function” analysis was conducted using IPA by incorporating its proprietary and Gene Ontology databases. Functional categories of numerous genes were identified, with significance determined at *p* < 0.05 (Table 2). Taurine induced notable alterations in multiple metabolic pathways, particularly in enhancing functions related to drug and lipid metabolism. Conversely, the downregulation of genes associated with cell death and carbohydrate metabolism was observed.

“Pathway” analysis was performed using IPA, which integrated its proprietary database and the Kyoto Encyclopedia of Genes and Genomes database, with significance set at *p* < 0.05. Taurine-induced alterations in genes related to drug metabolism pathways, such as the metabolism of xenobiotics by cytochrome p450, xenobiotic metabolism signaling, and aryl hydrocarbon receptor signaling. Taurine affects the genes associated with lipid metabolism, including arachidonic acid metabolism, linoleic acid metabolism, fatty acid metabolism, and steroid biosynthesis. Furthermore, genes involved in amino acid metabolism, such as tryptophan, glycine, serine, threonine, methionine, and lysine biosynthesis, were also influenced by taurine. Moreover, taurine-regulated genes related to glucose metabolism via insulin signaling and bile acid synthesis were also associated with cholesterol metabolism (Table 3).

### 3.4. Taurine Targeted the SHP as an Upstream Factor for Cholesterol Catabolism and Gluconeogenesis in Rats Fed a High Cholesterol Diet

**Figure 3:**
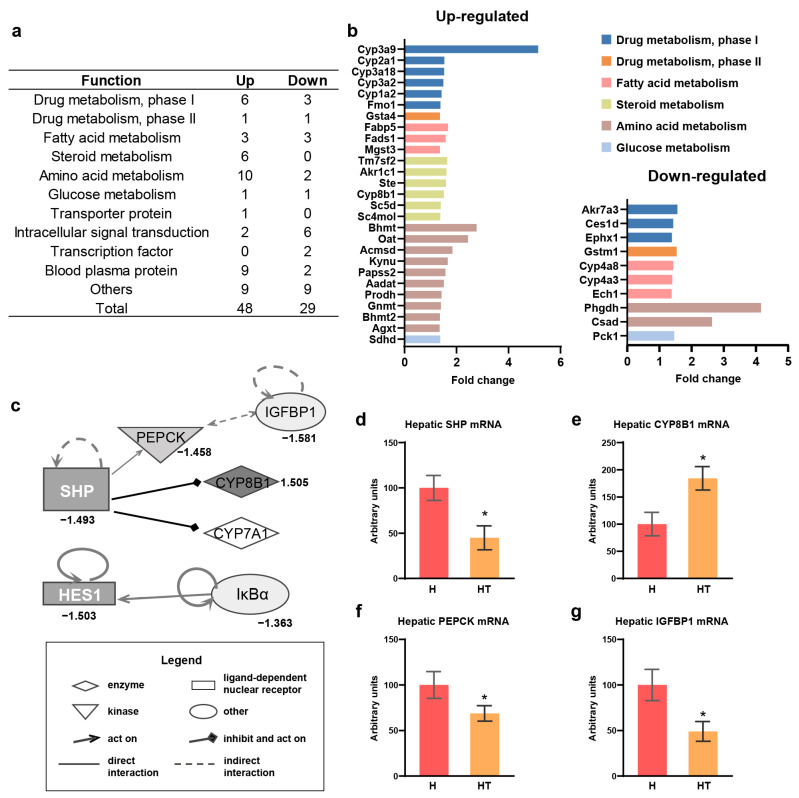
Taurine modulated multiple metabolisms by downregulating SHP gene expression in high cholesterol diet-fed rat liver. (**a**) The number of genes with a fold change threshold greater than 1.35 in rat liver between the high cholesterol plus taurine group and high cholesterol group; genes were filtered using IPA filter, specifying the species “Rat” and tissue “Liver”; (**b**) the fold change in gene expression across several functional categories; (**c**) effect of taurine on transcription factor related “network”; (**d**–**g**) hepatic SHP, CYP8B1, PEPCK, and IGFBP1 mRNA levels. (**c**) The numbers under the objects indicate fold induction regulated by taurine in microarray; CYP7A1 was upregulated by taurine and was detected by RT-qPCR; (**d**–**g**) H: high cholesterol group and HT: high cholesterol plus taurine group. Values represent the mean ± SEM, n = 6 of each group. The statistical results of the Student’s *t*-test are indicated. * These values differed significantly (*p* < 0.05) between the high cholesterol group and the high cholesterol plus taurine group.

To further investigate the molecular-level effects of taurine, the seventy-seven functions of the individual genes (Supplementary Table S2) were categorized into 11 distinct categories, including drug metabolism phases I and II, fatty acid metabolism, steroid metabolism, and amino acid metabolism (Figure 3a,b). Specifically, the taurine effect showed that genes from the CYP3A and CYP2A families were upregulated in phase I drug metabolism and that CYP8B1, which is involved in cholesterol metabolism, was upregulated in steroid metabolism (Figure 3b). Furthermore, genes related to glucose metabolism, such as phosphoenolpyruvate carboxykinase 1 (PEPCK, PCK1), were downregulated by taurine (Figure 3b).

Two transcription factors downregulated by taurine were hypothesized to regulate the metabolic pathway preceding the genes affected by taurine and, thus, were subjected to “network” analysis. Hes family bHLH transcription factor 1 (HES1) was found to be regulated by not only taurine but also NF-κB inhibitor alpha (IκBα) (Figure 3c). Additionally, the analysis indicated associations with the cholesterol metabolism enzyme CYP8B1 and gluconeogenesis enzyme PEPCK for the small heterodimer partner (SHP) (Figure 3c). Moreover, insulin-like growth factor binding protein 1 (IGFBP1) was linked to PEPCK and CYP7A1, whose gene expression increased with taurine, as confirmed by RT-qPCR, was incorporated into the network (Figure 2a and Figure 3c). The network illustrated the upregulation of cholesterol catabolism and downregulation of gluconeogenesis, which were attributed to the reduced expression of the SHP (Figure 3c). The above genes within the SHP network, exhibiting varying expression patterns according to microarray analysis, were selected for validation using RT-qPCR. Hepatic mRNA levels of the SHP, PEPCK, and IGFBP1 were decreased, whereas CYP8B1 mRNA levels were increased by taurine in the high cholesterol diet group, which was consistent with the results of the microarray analysis (Figure 3d–g).

### 3.5. Effect of Taurine on Gene Expression in Rat 3D-Primary Hepatocytes

The molecular mechanism underlying the effects of taurine in the rat liver involves inter-organ communication, including interactions with organs such as the intestine, heart, and brain [34]. To screen for direct target genes of taurine, we utilized the 3D-primary hepatocyte culture, which maintains hepatocyte differentiation.

Microarray analysis was performed on rat 3D-primary hepatocytes treated with dexamethasone, with or without 10 mM taurine supplementation (N and NT groups), on an EHS gel. Using the IPA filter species “rat” and tissue “liver”, the analysis identified 42 upregulated and 30 downregulated genes by taurine supplementation to the medium (Supplementary Table S3).

### 3.6. Taurine Direct Target Betaine Homocysteine Methyltransferase (BHMT) and Organic Anion Transporting Polypeptide 2 (OATP2) in Rat 3D-Primary Hepatocytes and Liver

**Figure 4:**
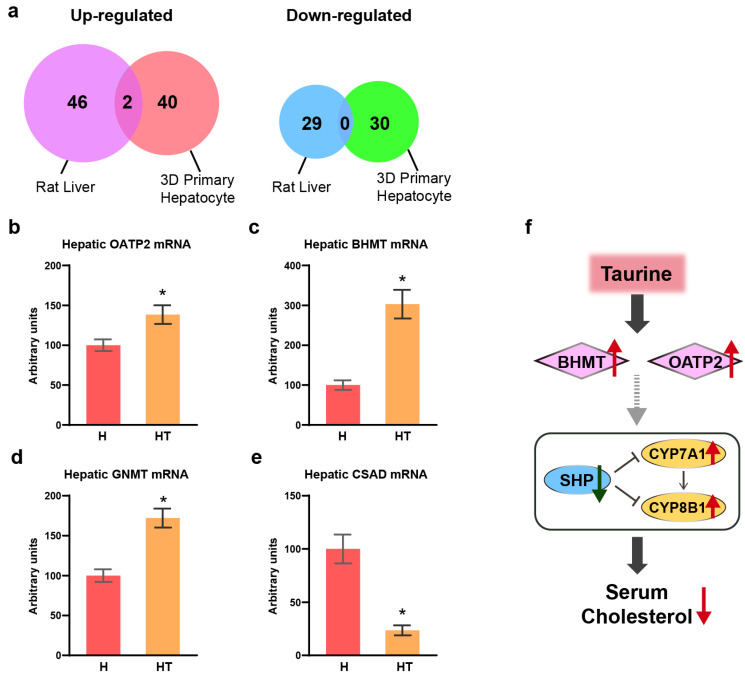
Taurine directly upregulated the hepatic expression of OATP2 and BHMT genes. (**a**) Venn diagrams showing the total number of genes regulated by taurine filtered using IPA filter, specifying the species “Rat” and tissue “Liver”; (**b**–**e**) hepatic OATP2, BHMT, GNMT, and CSAD mRNA levels in rat liver. (**f**) Schematic illustration of molecular mechanism: taurine directly enhances hepatic gene expression of BHMT and OATP2 associated with the amelioration of hypercholesterolemia and increase in CYP7A1 and CYP8B1 through the SHP in rats. H: high cholesterol group and HT: high cholesterol plus taurine group. (**b**–**e**) Values represent the mean ± SEM, n = 6 of each group. The statistical results of the Student’s *t*-test are indicated. * These values differed significantly (*p* < 0.05) between the high cholesterol group and the high cholesterol plus taurine group.

Venn diagram analysis revealed that only two genes, BHMT and OATP2 (also known as solute carrier organic anion transporter family 1A2, SLCO1A2), exhibited consistent upregulation in response to taurine treatment in both 3D-primary hepatocytes and the livers of rats fed a high cholesterol diet (Figure 4a). This effect was further confirmed by RT-qPCR, which showed that taurine increased hepatic BHMT and OATP2 mRNA levels in rats fed the high cholesterol diet (Figure 4b,c). Moreover, another methyltransferase involved in the methionine cycle, glycine N-methyltransferase (GNMT), showed an increased expression in response to taurine treatment (Figure 3b and Figure 4d). The enzyme downstream of the methionine cycle that regulates taurine endogenesis, cysteine sulfinic acid decarboxylase (CSAD), exhibited decreased gene expression upon taurine treatment (Figure 3b and Figure 4e).

## 4. Discussion

The concept that taurine deficiency is a pivotal marker of aging has sparked widespread interest in its effects on physiological functions and metabolism [6]. Cholesterol contributes to the normal functioning of vertebrates, whereas excess cholesterol accumulation in the blood is known to contribute to atherosclerotic cardiovascular diseases [35]. Specifically, in an ApoE-deficient mouse model, which exhibits severe hypercholesterolemia and atherogenic lesions similar to those found in humans, taurine supplementation inhibited the development of atherosclerotic lesions and showed protective potential against cardiovascular disease [13]. It has been previously reported that taurine ameliorates blood cholesterol accumulation in hypercholesterolemia and promotes cholesterol catabolism to bile acids by increasing CYP7A1 gene expression and activity [15,16,17,18]. However, the specific mechanism underlying the effect of taurine on CYP7A1 remains poorly understood, and many aspects of the potential of taurine in addressing hypercholesterolemia remain unexplored. Therefore, we conducted a transcriptomic analysis in the livers of rats fed a high cholesterol diet to thoroughly screen all genes regulated by taurine in vivo and then filter out the directly regulated and functional genes of taurine in combination with highly differentiated 3D-primary hepatocytes [28,29]. The objective of this study was to elucidate the molecular mechanisms involved, comprehensively explore their physiological functions, and identify the upstream regulator of CYP7A1, which is regulated by taurine.

We previously reported that taurine supplementation, in dosages ranging from 2.5 g to 50 g per kg of diet over a 2-week feeding period, can dose-dependently restore hypercholesterolemia induced by a high cholesterol diet [16]. Moreover, supplementation of 50 g taurine/kg diet increased the fecal excretion of cholesterol and bile acids, normalized serum total cholesterol, very-low-density lipoprotein cholesterol, and high-density lipoprotein cholesterol concentrations, and reduced lipid cholesterol levels in rats [16,36]. This study validated the ameliorative effects of taurine on dietary cholesterol-induced hypercholesterolemia under the same conditions. The dietary taurine facilitated cholesterol catabolism by improving CYP7A1, thereby restoring serum cholesterol levels to normal and ameliorating hypercholesterolemia induced by high cholesterol diet (Figure 1d). In addition, taurine increased the transcription rate of CYP7A1, implying that the regulatory effect of taurine was achieved through transcription factors and at the transcriptional level (Figure 2).

The discussion on the biological functions and pathways of taurine in the liver of rats fed a high cholesterol diet highlights its multifaceted role in metabolism, encompassing drug metabolism and three macronutrients: carbohydrates, lipids, and proteins (Table 2 and Table 3). In particular, taurine upregulated the expression of genes of the cytochrome p450 family, which catalyzes the first phase of drug and xenobiotic metabolism (Figure 3b). This indicates the potential of combining taurine with medication. The protective role of taurine against hepatotoxicity induced by doxorubicin, tamoxifen, or methotrexate is well documented [37,38,39]. Additionally, taurine increased the expression of CYP8B1, a key catabolic enzyme for the conversion of cholesterol to cholic acid, indicating the blood cholesterol-lowering effect of taurine involves CYP7A1 and CYP8B1, including the 7α-hydroxylation of cholesterol and subsequent primary bile acid synthesis (Figure 2b and Figure 3e) [40]. Taurine also modulated glucose metabolism through insulin receptor signaling (Table 3), resulting in the downregulation of PEPCK (Figure 3b), which catalyzes the initial step in hepatic gluconeogenesis [41]. Moreover, the reduction in PEPCK activity correlates with a significant decrease in the citric acid cycle activity [42], which plays a central role in fat and cholesterol metabolism. Consequently, the decrease in crucial precursors such as acetyl-CoA impacts the synthesis of fatty acid and cholesterol.

Furthermore, network analysis revealed that taurine intake leads to decreased SHP expression, a transcription factor, resulting in positive regulation of PEPCK and negative regulation of CYP7A1 and CYP8B1 (Figure 3c). This suggests the SHP is a molecular target for taurine, influencing cholesterol catabolic pathway and gluconeogenesis, acting upstream of CYP7A1, CYP8B1, and PEPCK [43,44,45]. The findings, confirmed through RT-qPCR (Figure 3d–f), imply that taurine may lower serum cholesterol by modulating SHP, thereby inducing CYP7A1 and CYP8B1 expression.

It is well established that gene expression in the liver is highly influenced by other organs, such as the intestine, brain, and heart, within the inter-organ communication network [34]. Specifically, the amelioration of cholesterol accumulation may be attributed not only to the role of CYP7A1 in the liver but also to the effects of taurine-conjugated bile acids in the intestine, as well as its neuroprotective effects, which subsequently influence metabolism in other organs. To identify the direct target genes of taurine in the liver, we conducted a 3D-primary hepatocyte culture that closely resembles the in vivo natural system and operates independently of other organs.

However, in the 3D-primary hepatocyte culture experiment [28,29], SHP, CYP7A1, and CYP8B1 gene expression were not regulated by taurine treatment (Figure 4a, Table S3). From the Venn diagram analysis, we found that only BHMT and OATP2 gene expression was upregulated by taurine in both rat 3D-primary hepatocytes and rat livers (Figure 4b,c, Table S3). OATP2 is a transporter involved in the absorption and elimination of bile acid (especially taurocholate) or other organic anions such as arachidonate and its metabolome prostaglandins and thromboxane from blood in rats [46]. The promotion of bile acid uptake by the liver is thought to lead to upregulation of the SHP and suppression of cholesterol conversion to bile acids [47]. However, our results suggest a contrary effect of OATP2 on SHP. This raises the possibility that OATP2 is not responsible for bile acid transport into the liver. Instead, the arachidonate metabolome, which is known as the conserved regulator of cholesterol metabolism, is transported to the liver via OATP2, thereby inhibiting the SHP [48]. Therefore, the exact modulation of the SHP and cholesterol catabolism by OATP2 under the action of taurine needs to be explored in subsequent studies. BHMT is a thiomethyltransferase that contributes to the production of methionine from homocysteine and has a well-established relationship with atherosclerosis [49]. Given that taurine treatment increased BHMT gene expression in the rat liver and rat 3D-primary hepatocytes, taurine is suggested to have a direct anti-atherosclerotic effect via gene expression (Figure 4c, Tables S2 and S3). Additionally, it has been reported that the betaine-BHMT system is involved in the liver induced by bile acid accumulation and that this protection is specific to homocysteine-induced endoplasmic reticulum (ER) stress and cell death [50]. Bioinformatic analysis of rat livers suggested that taurine can downregulate cell death and homocysteine-inducible ER protein with ubiquitin-like domain 1 gene expression (Table 2 and Table S2). Thus, BHMT may act as a direct target of taurine during ER stress and bile acid homeostasis. Overall, taurine-regulated transcription factors should directly increase the gene expression of BHMT and OATP2 in the SHP, CYP7A1, and CYP8B1 networks, leading to a reduction in serum cholesterol levels in rats.

Moreover, many genes involved in amino acid metabolism were upregulated by taurine, with another S-adenosylmethionine-dependent methyltransferase in the methionine cycle, GNMT, showing a significant increase in the rat liver after taurine supplementation (Figure 3b) [51,52]. With the increasing recognition of the importance of methionine cycle-related gene expression, especially GNMT and BHMT, in the epigenetic modification of DNA methylation, taurine may also emerge as a potential nutrient contributing to epigenetic changes [53]. Moreover, taurine homeostasis in the liver is maintained by inhibiting endogenous synthesis by suppressing the expression of the key enzyme CSAD when taurine is supplemented in the diet [54].

Overall, we have identified the extensive physiological effects of taurine in the livers of rats fed a high cholesterol diet. Additionally, we have preliminarily determined the regulatory genes (BHMT and OATP2) directly regulated by taurine in hepatocytes. However, our findings are currently limited to mRNA-level regulatory effects. The precise regulatory mechanisms, such as the roles of transcription factors and response elements, remain to be elucidated and will be the focus of our future study. These findings highlight the necessity for further investigation into the specific mechanisms through which taurine exerts its regulatory effects.

## 5. Conclusions

Taurine contributes to blood cholesterol lowering by inducing the CYP7A1 and CYP8B1 expression through SHP regulation. Moreover, BHMT and OATP2 have been identified as direct targets of taurine in the liver. Overall, taurine exerts stimulatory effects on drug and lipid metabolism, inhibitory effects on gluconeogenesis, and anti-atherosclerotic effects. The elucidation of the molecular action of taurine (shown in Figure 4f) in this study allowed for a more direct assessment of its effects. However, further investigation into the direct taurine modulation requires greater attention in future research.

## Figures and Tables

**Table 1:** Composition of the experimental diets.

Ingredient (g/kg Diet)	Control (C)	Taurine (CT)	High Cholesterol (H)	High Cholesterol Plus Taurine (HT)
Casein ^1^	200	200	200	200
Vitamin mixture ^2^	10	10	10	10
Mineral mixture ^3^	35	35	35	35
Choline chloride	2	2	2	2
Corn oil ^4^	50	50	50	50
Cellulose	50	50	50	50
Starch	435	402	427	394
Sucrose	218	201	213.5	196.5
Cholesterol	-	-	10	10
Sodium cholate	-	-	2.5	2.5
Taurine	-	50	-	50

^1^ Casein (#9000-71-9, Sigma-Aldrich Japan, Tokyo, Japan); ^2^ vitamin mixture (AIN93-VX, CLEA Japan, Inc., Tokyo, Japan); ^3^ mineral mixture (AIN93G-MX, CLEA Japan, Inc., Tokyo, Japan); ^4^ corn oil (J-OIL MILLS, Inc., Tokyo, Japan).

**Table 2:** “Function” analysis with Ingenuity Pathway Analysis (IPA) of taurine-changed gene in high cholesterol diet rat liver ^1^.

Functional Category ^2^	*p*-Value
**Regulated by taurine**	
Drug Metabolism	3.42 × 10^−6^
Endocrine System Development and Function	3.42 × 10^−6^
Lipid Metabolism	3.42 × 10^−6^
Small Molecule Biochemistry	3.42 × 10^−6^
Molecular Transport	9.54 × 10^−3^
Cellular Function and Maintenance	1.52 × 10^−2^
Cell Death	3.02 × 10^−2^
Carbohydrate Metabolism	4.50 × 10^−2^
Hepatic System Disease	4.50 × 10^−2^
**Upregulated by taurine ^3^**	
Drug Metabolism	6.59 × 10^−7^
Endocrine System Development and Function	6.59 × 10^−7^
Lipid Metabolism	6.59 × 10^−7^
Small Molecule Biochemistry	6.59 × 10^−7^
Molecular Transport	3.32 × 10^−3^
Hepatic System Disease	2.64 × 10^−2^
Cancer	3.50 × 10^−2^
Gastrointestinal Disease	3.50 × 10^−2^
**Downregulated by taurine ^3^**	
Cellular Function and Maintenance	6.36 × 10^−3^
Cell Death	1.27 × 10^−2^
Carbohydrate Metabolism	1.90 × 10^−2^
Molecular Transport	1.90 × 10^−2^

^1^ The analysis was performed using IPA with Gene Ontology (GO) database and IPA database. ^2^ The list of categories was filtered using IPA filter, specifying the species “Rat” and tissue “Liver”, with a significance threshold of *p* < 0.05. ^3^ Upregulated and downregulated categories were analyzed based on significant upregulated and downregulated genes, respectively.

**Table 3:** “Pathway” analysis with IPA of taurine-changed gene in high cholesterol diet rat liver ^1^.

Category ^2^	*p*-Value	Ratio (%)
Metabolism of Xenobiotics by Cytochrome P450	7.76 × 10^−9^	15.3
LPS/IL-1 Mediated Inhibition of RXR Function	2.34 × 10^−8^	9.79
Tryptophan Metabolism	9.55 × 10^−7^	11.0
Arachidonic Acid Metabolism	2.04 × 10^−6^	11.7
Linoleic Acid Metabolism	1.15 × 10^−5^	13.5
PXR/RXR Activation	1.91 × 10^−5^	12.5
Fatty Acid Metabolism	3.63 × 10^−5^	9.52
Acute Phase Response Signaling	1.45 × 10^−4^	6.92
Glycine, Serine, and Threonine Metabolism	3.98 × 10^−4^	11.9
Xenobiotic Metabolism Signaling	1.29 × 10^−3^	5.14
NRF2-Mediated Oxidative Stress Response	2.24 × 10^−3^	5.83
Methionine Metabolism	2.75 × 10^−3^	15.8
Glutathione Metabolism	3.31 × 10^−3^	9.76
Lysine Biosynthesis	6.03 × 10^−3^	25.0
Complement System	6.17 × 10^−3^	12.0
Coagulation System	8.51 × 10^−3^	10.7
Hepatic Cholestasis	1.51 × 10^−2^	5.21
Sulfur Metabolism	1.62 × 10^−2^	15.4
Pantothenate and CoA Biosynthesis	1.62 × 10^−2^	15.4
Aryl Hydrocarbon Receptor Signaling	1.86 × 10^−2^	4.95
Inositol Metabolism	2.69 × 10^−2^	6.98
Biosynthesis of Steroids	3.02 × 10^−2^	11.1
Insulin Receptor Signaling	3.31 × 10^−2^	5.0

^1^ The analysis was performed using IPA with the Kyoto Encyclopedia of Genes and Genomes (KEGG) database and IPA database. ^2^ The list of categories was filtered using IPA filter, specifying the species “Rat” and tissue “Liver”, with a significance threshold of *p* < 0.05.

## Data Availability

Data is contained within the article and Supplementary Materials.

## Supplementary Materials

The following supporting information can be downloaded at: https://www.mdpi.com/article/10.3390/antiox13080990/s1, Table S1: Primers for qPCR; Table S2: Gene regulated in the liver of the rats fed a high cholesterol supplemented with taurine as compared with control rats fed a cholesterol die; and Table S3: Gene regulated in rat primary hepatocyte supplemented with taurine.
