# Supplementary material for: Direct Molecular Action of Taurine on Hepatic Gene Expression Associated with the Amelioration of Hypercholesterolemia in Rats
Source: Antioxidants (Basel). 2024 Aug 14;13(8):990. doi: 10.3390/antiox13080990 (PMC11351134; doi:10.3390/antiox13080990)
Supplement: Supplementary file 1 [file antioxidants-13-00990-s001.zip › antioxidants-3114585-supplementary.pdf]

Supplementary

Table S1. Primers for qPCR.

Gene	Accession number	Forward primer (5'→3')	Reverse primer (5'→3')
APOE	NM_138828	TGAACCGCTTCTGGGATTAC	TGTGTGACTTGGGAGCTCTG
BHMT	NM_030850	TGGGCAGAAGGTCAATGAAG	AGCTGAGGTAGGAAGGTGTC
CSAD	NM_021750	ATTTCACCCTGATGGCTGAC	CTACGACAATCCCAAACACG
CYP7A1	NM_012942	TGTGTGAGGGACCAGGTCTCT	AGCTCAAAAGGTTGGAGGA
CYP7A1 (pre-mature)	NC_086023	GAGTAGTATTTGGGAGGGATC	TGAATGTGTGTTTGCTGAGGC
CYP8B1	NM_031241	GCCTCTTCCACTTCTGCTAC	ACTTCCTGAACAGCTCATCG
GNMT	NM_017084	GCTGAAATACGCACTGAAGG	ACTTGTCAAAGGCTGGCTCC
IGFBP1	NM_013144	GCCATTAGCACCTACAGCAG	AGGGCTCCTTCCATTTCTTG
OATP2	NM_131906	GCTTCAATGGCTTAGTGTTC	AACTTTCTATCTGGTCCCTC
PEPCK	NM_198780	AACTGTTGGCTGGCTCTCAC	TCTGCTCTTGGGTAATGATG
SHP	NM_057133	CAGCTTGGATTTCCTCGGTTT	GTCTGGAGGAATTCTGCCCTG

Table S2. Gene regulated in the liver of the rats fed a high cholesterol supplemented with taurine as compared with control rats fed a cholesterol diet [1]

Agilent	Symbol	Entrez Gene Name	Fold change
**Up-regulated**			
A_42_P547659	Cyp3a9	cytochrome P450, family 3, subfamily a, polypeptide 9	5.14
A_42_P662561	Bhmt	betaine-homocysteine S-methyltransferase	2.77
A_43_P12236	Oat	ornithine aminotransferase	2.44
A_42_P581804	Igfbp2	insulin-like growth factor binding protein 2	2.09
A_43_P13249	Acmsd	aminocarboxymuconate semialdehyde decarboxylase	1.84
A_43_P18325	Nnmt	nicotinamide N-methyltransferase	1.72
A_42_P708480	Slco1a2	solute carrier organic anion transporter family, member 1a2	1.68
A_42_P766084	Fabp5	fatty acid binding protein 5	1.67
A_43_P14915	C6	complement C6	1.67
A_42_P703548	Kynu	kynureninase	1.66
A_42_P814765	Tm7sf2	transmembrane 7 superfamily member 2	1.64
A_43_P17435	Akr1c1	aldo-keto reductase family 1, member C1	1.61
A_42_P753935	Ste	estrogen sulfotransferase	1.59
A_42_P616434	Fads1	fatty acid desaturase 1	1.58
A_42_P513050	Papss2	3'-phosphoadenosine 5'-phosphosulfate synthase 2	1.57
A_43_P12028	Rgs3	regulator of G-protein signaling 3	1.57
A_43_P12752	Orm1	orosomucoid 1	1.54
A_43_P16979	Psmc6	proteasome 26S subunit, ATPase 6	1.54
A_42_P654578	Cyp2a1	cytochrome P450, family 2, subfamily a, polypeptide 1	1.53
A_42_P679814	Cyp3a18	cytochrome P450, family 3, subfamily a, polypeptide 18	1.52
A_42_P594613	Dusp6	dual specificity phosphatase 6	1.52
A_42_P473074	Cyp8b1	cytochrome P450 family 8 subfamily B member 1	1.51
A_42_P682853	Aadat	aminoadipate aminotransferase	1.51
A_42_P584188	Cyp3a2	cytochrome P450, family 3, subfamily a, polypeptide 2	1.49
A_43_P11448	Cp	ceruloplasmin	1.49
A_42_P489764	Gjb2	gap junction protein, beta 2	1.46
A_43_P16690	Uqcrc2	ubiquinol cytochrome c reductase core protein 2	1.44
A_43_P11454	Cyp1a2	cytochrome P450, family 1, subfamily a, polypeptide 2	1.42
A_43_P16750	Prodh	proline dehydrogenase	1.42
A_42_P581158	Eif4a2	eukaryotic translation initiation factor 4A2	1.42
A_42_P774912	C4bpb	complement component 4 binding protein, beta	1.41
A_42_P502960	Gnmt	glycine N-methyltransferase	1.40

A_43_P23175	Fga	fibrinogen alpha chain	1.40
A_43_P16256	Fgb	fibrinogen beta chain	1.40
A_42_P554971	Sc5d	sterol-C5-desaturase	1.39
A_42_P644641	Fmo1	flavin containing dimethylaniline monoxygenase 1	1.38
A_42_P504124	Psmd12	proteasome 26S subunit, non-ATPase 12	1.38
A_43_P13088	Sc4mol	sterol-C4-methyl oxidase	1.37
A_42_P560288	Sdhd	succinate dehydrogenase complex subunit D	1.37
A_43_P15364	C1s	complement C1s	1.37
A_42_P773816	Cd1d1	CD1d1 molecule	1.37
A_42_P531078	Gsta4	glutathione S-transferase, alpha 4	1.36
A_42_P522857	Mgst3	microsomal glutathione S-transferase 3	1.36
A_42_P581488	Bhmt2	betaine-homocysteine S-methyltransferase 2	1.36
A_43_P11464	Fgg	fibrinogen gamma chain	1.36
A_42_P704269	Dpyd	dihydropyrimidine dehydrogenase	1.36
A_43_P12449	Agxt	alanine--glyoxylate aminotransferase	1.35
A_43_P11818	Crp	C-reactive protein	1.35
**Down-regulated**			
A_43_P12981	Trip10	thyroid hormone receptor interactor 10	1.35
A_42_P544487	Nfkbia	NFKB inhibitor alpha	1.36
A_43_P10997	Ppp1r3c	protein phosphatase 1, regulatory subunit 3C	1.36
A_43_P16675	Mmp15	matrix metallopeptidase 15	1.37
A_42_P563285	Ech1	enoyl-CoA hydratase 1	1.38
A_43_P11584	Ephx1	epoxide hydrolase 1	1.39
A_43_P15784	Cyp4a3	cytochrome P450, family 4, subfamily a, polypeptide 3	1.40
A_43_P13297	Mt1	metallothionein 1	1.40
A_42_P489710	Polm	DNA polymerase mu	1.41
A_43_P11634	Gfra1	GDNF family receptor alpha 1	1.42
A_42_P570313	Ces1d	carboxylesterase 1D	1.43
A_42_P735495	Cyp4a8	cytochrome P450, family 4, subfamily a, polypeptide 8	1.43
A_42_P805300	Gpx1	glutathione peroxidase 1	1.43
A_43_P16122	Adcy7	adenylate cyclase 7	1.44
A_42_P574741	H1f0	H1 histone family, member 0	1.44
A_42_P511615	Ppcs	phosphopantothenoylcysteine synthetase	1.45
A_42_P589366	Pck1	phosphoenolpyruvate carboxykinase 1	1.46
A_43_P15282	Sgk1	serum/glucocorticoid regulated kinase 1	1.46
A_43_P13041	Nr0b2	nuclear receptor subfamily 0, group B, member 2	1.49
A_43_P14926	Hes1	hes family bHLH transcription factor 1	1.50
A_43_P12445	Alas1	5'-aminolevulinate synthase 1	1.52
A_43_P11776	Gstm1	glutathione S-transferase mu 1	1.54
A_43_P11754	Akr7a3	Aldo-keto reductase family 7 member A3	1.56
A_42_P830405	Herpud1	homocysteine inducible ER protein with ubiquitin-like domain 1	1.57

A_42_P549562	Igfbp1	insulin-like growth factor binding protein 1	1.58
A_43_P14443	Car8	carbonic anhydrase 8	1.72
A_43_P12114	Csad	cysteine sulfinic acid decarboxylase	2.64
A_42_P710738	Phgdh	phosphoglycerate dehydrogenase	4.16
A_43_P18493	Rapgef2	Rap guanine nucleotide exchange factor 2	4.87

Note: The list of genes was filtered using IPA filter, specifying the species “Rat” and tissue “Liver”, with a fold change threshold greater than 1.35.

Table S3. Gene regulated in rat primary hepatocyte supplemented with taurine [1]

Affymetrix	Symbol	Entrez Gene Name	Log2 Ratio
1389539_at	APPL1	adaptor protein, phosphotyrosine interaction, PH domain and leucine zipper containing 1	0.9
1385043_at	INADL	InaD-like (Drosophila)	0.9
1379499_at	LTB	lymphotoxin beta (TNF superfamily, member 3)	0.9
1395337_at	TJP1	tight junction protein 1 (zona occludens 1)	0.9
1369202_at	MX1	myxovirus (influenza virus) resistance 1, interferon-inducible protein p78 (mouse)	0.8
1388239_at	PER3	period homolog 3 (Drosophila)	0.8
1377832_at	PLK4	polo-like kinase 4 (Drosophila)	0.8
1393705_at	CD2AP	CD2-associated protein	0.7
1389871_at	GOT2	glutamic-oxaloacetic transaminase 2, mitochondrial (aspartate aminotransferase 2)	0.7
1371033_at	HLA-DQB1	major histocompatibility complex, class II, DQ beta 1	0.7
1368086_a_at	LSS	lanosterol synthase (2,3-oxidosqualene-lanosterol cyclase)	0.7
1384962_at	MED1	mediator complex subunit 1	0.7
1392643_at	RAB5B	RAB5B, member RAS oncogene family	0.7
1375138_at	TIMP3	TIMP metallopeptidase inhibitor 3	0.7
1370485_a_at	BCL2L1	BCL2-like 1	0.6
1368850_at	CSNK1G3	casein kinase 1, gamma 3	0.6
1387242_at	EIF2AK2	eukaryotic translation initiation factor 2-alpha kinase 2	0.6
1369703_at	EPAS1	endothelial PAS domain protein 1	0.6
1370750_a_at	IL1R1	interleukin 1 receptor, type I	0.6
1373975_at	INMT	indolethylamine N-methyltransferase	0.6
1375371_at	RANBP2	RAN binding protein 2	0.6
1385194_at	RB1CC1	RB1-inducible coiled-coil 1	0.6
1391543_at	RIPK1	receptor (TNFRSF)-interacting serine-threonine kinase 1	0.6
1387094_at	SLCO1A2	solute carrier organic anion transporter family, member 1A2	0.6
1391404_at	STAG2	stromal antigen 2	0.6
1387983_at	THRB	thyroid hormone receptor, beta (erythroblastic leukemia viral (v-erb-a) oncogene homolog 2, avian)	0.6
1390059_at	USP25	ubiquitin specific peptidase 25	0.6
1369526_at	ACADSB	acyl-Coenzyme A dehydrogenase, short/branched chain	0.5
1370955_at	ADAM10	ADAM metallopeptidase domain 10	0.5
1392864_at	ARHGAP5	Rho GTPase activating protein 5	0.5
1387665_at	BHMT	betaine-homocysteine methyltransferase	0.5
1369186_at	CASP1	caspase 1, apoptosis-related cysteine peptidase (interleukin 1, beta, convertase)	0.5
1387316_at	CXCL2	chemokine (C-X-C motif) ligand 2	0.5
1369220_at	DNM1L	dynamin 1-like	0.5
1382040_at	EPRS	glutamyl-prolyl-tRNA synthetase 1	0.5
1374422_at	ERBB2IP	erbb2 interacting protein	0.5

	(includes EG:55914)		
1393151_at	ETNK1	ethanolamine kinase 1	0.5
1371942_at	GSTT3	glutathione S-transferase, theta 3	0.5
1386965_at	LPL	lipoprotein lipase	0.5
1388245_a_at	NRG1	neuregulin 1	0.5
1377833_at	RIF1	RAP1 interacting factor homolog (yeast)	0.5
1394059_s_at	YME1L1	YME1-like 1 (S.cerevisiae)	0.5
1380530_at	MAML2	mastermind-like 2 (Drosophila)	-3.7
1369570_at	SULT2A1	sulfotransferase family, cytosolic,2A, dehydroepiandrosterone (DHEA)-preferring, member1	-1.5
1393555_at	HSP90AB1	heat shock protein 90kDa alpha (cytosolic), class B member 1	-0.9
1388096_at	KITLG	KIT ligand	-0.9
1369825_at	MMP2	matrix metallopeptidase 2 (gelatinase A, 72kDa gelatinase, 72kDa type IV collagenase)	-0.9
1370789_a_at	PRLR	prolactin receptor	-0.9
1376835_at	NFKBIE	nuclear factor of kappa light polypeptide gene enhancer in B-cells inhibitor, epsilon	-0.8
1371263_a_at	CAMK2D	calcium/calmodulin-dependent protein kinase II delta	-0.7
1387062_a_at	CHEK1	CHK1 checkpoint homolog (S. pombe)	-0.7
1369292_at	HSD17B1	hydroxysteroid (17-beta) dehydrogenase 1	-0.7
1386989_at	S1PR2	sphingosine-1-phosphate receptor 2	-0.7
1370574_a_at	SIRPA	signal-regulatory protein alpha	-0.7
1387135_at	ADAM15	ADAM metallopeptidase domain 15	-0.6
1370832_at	CCL4	chondroitin sulfate synthase 3	-0.6
1386783_at	CHSY3	chemokine (C-C motif) ligand 4	-0.6
1385439_x_at	DHX58	DEXH (Asp-Glu-X-His) box polypeptide 58	-0.6
1371414_at	GSN	gelsolin (amyloidosis, Finnish type)	-0.6
1396090_at	RNF150	ring finger protein 150	-0.6
1398279at	TRPV5	transient receptor potential cation channel, subfamily V, member 5	-0.6
1398624_x_at	TUBB4	tubulin, beta 4	-0.6
1381931_at	ABLIM1	actin binding LIM protein 1	-0.5
1399096_at	ADD3	adducin 3 (gamma)	-0.5
1390325_at	CD38	CD38 molecule	-0.5
1392854_at	DDX6	DEAD (Asp-Glu-Ala-Asp) box polypeptide 6	-0.5
1369608_at	FGF16	fibroblast growth factor 16	-0.5
1371032_at	NID1	nidogen 1	-0.5
1374235_at	RCAN2	regulator of calcineurin 2	-0.5
1390707_at	RGS10	regulator of G-protein signaling 10	-0.5
1392231_at	STK4	serine/threonine kinase 4	-0.5
1392382_at	TGFB2	transforming growth factor, beta 2	-0.5

Note: The list of genes was filtered using IPA filter, specifying the species “Rat” and tissue “Liver”, with a Log2 ratio threshold greater than 0.5 or less than -0.5.
